# Participant recruitment, baseline characteristics and at-home-measurements of cardiometabolic risk markers: insights from the Supreme Nudge parallel cluster-randomised controlled supermarket trial

**DOI:** 10.1186/s13063-023-07157-8

**Published:** 2023-03-02

**Authors:** Josine M. Stuber, Beryl A. C. E. van Hoek, Anne L. Vos, Edith G. Smit, Jeroen Lakerveld, Joreintje D. Mackenbach, Joline W. J. Beulens, on behalf of the SUPREME NUDGE consortium, Jody C. Hoenink, Femke Rutters, Wilma E. Waterlander, Denise T. D. de Ridder, Marleen Gillebaart, Stephanie Blom, Femke E. de Boer, Gert-Jan de Bruijn, Michel C. A. Klein, Jacqueline E. W. Broerse, Tjerk-Jan Schuitmaker-Warnaar, Cédric N. H. Middel, Yvonne T. van der Schouw, Ivonne Sluijs, Marjolein C. Harbers, Elizabeth Velema

**Affiliations:** 1 Amsterdam UMC location Vrije Universiteit Amsterdam, Epidemiology and Data Science, De Boelelaan, 1117 Amsterdam, The Netherlands; 2 Amsterdam Public Health, Amsterdam, The Netherlands; 3 Amsterdam UMC location Vrije Universiteit Amsterdam, Department of General Practice, De Boelelaan, 1117 Amsterdam, The Netherlands; 4 Amsterdam School of Communication Research, University of Amsterdam, Nieuwe Achtergracht 166, 1018 WV Amsterdam, The Netherlands; 5 Julius Center for Health Sciences and Primary Care, University Medical Center Utrecht, Utrecht University, Universiteitsweg, 100 Utrecht, The Netherlands

**Keywords:** SES, Prevention, Intervention, Diet, Physical activity

## Abstract

**Background:**

Recruiting participants for lifestyle programmes is known to be challenging. Insights into recruitment strategies, enrolment rates and costs are valuable but rarely reported. We provide insight into the costs and results of used recruitment strategies, baseline characteristics and feasibility of at-home cardiometabolic measurements as part of the Supreme Nudge trial investigating healthy lifestyle behaviours. This trial was conducted during the COVID-19 pandemic, requiring a largely remote data collection approach. Potential sociodemographic differences were explored between participants recruited through various strategies and for at-home measurement completion rates.

**Methods:**

Participants were recruited from socially disadvantaged areas around participating study supermarkets (*n* = 12 supermarkets) across the Netherlands, aged 30–80 years, and regular shoppers of the participating supermarkets. Recruitment strategies, costs and yields were logged, together with completion rates of at-home measurements of cardiometabolic markers. Descriptive statistics are reported on recruitment yield per used method and baseline characteristics. We used linear and logistic multilevel models to assess the potential sociodemographic differences.

**Results:**

Of 783 recruited, 602 were eligible to participate, and 421 completed informed consent. Most included participants were recruited via letters/flyers at home (75%), but this strategy was very costly per included participant (89 Euros). Of paid strategies, supermarket flyers were the cheapest (12 Euros) and the least time-invasive (< 1 h). Participants who completed baseline measurements (*n* = 391) were on average 57.6 (SD 11.0) years, 72% were female and 41% had high educational attainment, and they often completed the at-home measurements successfully (lipid profile 88%, HbA1c 94%, waist circumference 99%). Multilevel models suggested that males tended to be recruited more often via word-of-mouth (OR_females_ 0.51 (95%CI 0.22; 1.21)). Those who failed the first attempt at completing the at-home blood measurement were older (*β* 3.89 years (95% CI 1.28; 6.49), whilst the non-completers of the HbA1c (*β* − 8.92 years (95% CI − 13.62; − 4.28)) and LDL (*β* − 3.19 years (95% CI − 6.53; 0.09)) were younger.

**Conclusions:**

Supermarket flyers were the most cost-effective paid strategy, whereas mailings to home addresses recruited the most participants but were very costly. At-home cardiometabolic measurements were feasible and may be useful in geographically widespread groups or when face to face contact is not possible.

**Trial registration:**

Dutch Trial Register ID NL7064, 30 May 2018, https://trialsearch.who.int/Trial2.aspx?TrialID=NTR7302

**Supplementary Information:**

The online version contains supplementary material available at 10.1186/s13063-023-07157-8.

## Background

Cardiometabolic diseases (CMD), including type 2 diabetes and cardiovascular diseases, pose a major individual and societal burden [1, 2]. Differences in prevalence of CMD and lifestyle behaviours have been associated with socioeconomic position (SEP), with a higher CMD burden for individuals with a low SEP [3]. It is well established that maintaining healthy lifestyle behaviours reduces CMD risk, but this is not straightforward for most individuals. For example, population surveys on dietary patterns show that at least half of Dutch adults do not adhere to dietary guidelines and even less than 25% consume enough fruit and vegetables [4], despite the widespread availability of information on the composition of a healthy diet.

It is clear that solely providing information on a healthy lifestyle is not sufficient to change behaviour, as many other individual-level factors than knowledge affect lifestyle behaviour, including motivations, beliefs, personal resources and preferences [5, 6]. In turn, these factors are driven by more ‘upstream determinants’ in the social-cultural environment (e.g. cultural beliefs on what is healthy), the built environment (e.g. availability of healthy food outlets), the economic environment (e.g. food prices) and the policy environment (e.g. health-promotion campaigns) [6–12]. Healthy lifestyle programmes that are designed to address these individual-level and upstream determinants could contribute to a CMD risk reduction and reduce health inequalities.

The effectiveness of lifestyle programmes is usually evaluated by randomised controlled trials, for which the inclusion of sufficient study participants is paramount. Low inclusion rates can result in a lack of statistical power to evaluate study findings [13, 14], leading to a waste of research funding. However, including a sufficient number of participants is known to be challenging. Well-designed recruitment strategies which are tailored to the target group are, therefore, crucial. Tailoring can be based on sociodemographic, sociocultural and socioeconomic characteristics and the developed recruitment strategy should match the study type and its design [15].

The combination of active and passive recruitment strategies is often used. Examples are the use of flyers, email invites or social media posts (all passive strategies) and face-to-face recruitment at the location of the target population (active strategy) [16–19]. Passive recruitment strategies are reported to be more cost-effective in the recruitment of participants for healthy lifestyle programmes than active strategies [18–20]. Active recruitment strategies, however, seem most effective in reaching those who would be likely to benefit most from a lifestyle programme [19, 20]. For instance, Smit et al. (2021) showed that of all participants enrolled in their trial, the healthiest participants in terms of physical activity guideline adherence were recruited via passive strategies such as personal letters/flyers, social media and word-of-mouth. In contrast, those with lower physical activity guideline adherence were more often recruited via health professional referrals (active strategy) [19].

Previous studies show that adults with a low SEP are a diverse group for whom various barriers exist to participate in healthy lifestyle programmes. Also, response rates are often lower than for those with a high SEP [21–24]. Examples of barriers to participation are psychosocial barriers, such as having other priorities or being sceptical, study-related challenges such as overly complex study materials and other reasons such as lack of motivation [21], family issues or financial constraints [25]. As such, it is important to account for potential barriers among the target group prior to recruitment (e.g. by providing easy-to-understand recruitment materials). In addition, when aiming to reach a diverse study population, it is likely important to use a variety of recruitment strategies [21, 25]. Yet, it is unclear which strategies can be most (cost)effective to reach a study sample consisting of a variety of individuals, including for example individuals with a low SEP.

Insights from projects into recruitment strategies and costs, participant enrolment rates and characteristics of the included study sample are valuable to learn from and for planning of new projects. However, detailed recruitment information and especially insights into costs and yield in terms of participant characteristics are rarely reported. We conducted a real-life trial—the Supreme Nudge trial—that aimed to promote healthy lifestyle behaviours within socially disadvantaged neighbourhoods in the Netherlands [26, 27]. We focused on healthy food choices via the implementation of nudging and pricing strategies in real-life supermarkets and on healthy walking behaviours via a smartphone coaching app. This trial was conducted during the COVID-19 pandemic, which required a predominantly remote recruitment and data collection approach. This paper aims to provide insight into the costs, feasibility and results of used recruitment strategies, the baseline characteristics of the included study sample and the feasibility of at-home measurement of cardiometabolic risk markers. Furthermore, we explored potential sociodemographic differences in the most effective recruitment strategies and completion of the at-home measurements of cardiometabolic risk makers.

## Methods

### The Supreme Nudge trial

Data presented in this paper are based on the recruitment and baseline measurement of the Supreme Nudge parallel cluster-randomised controlled trial, of which the study protocol was previously published [26]. Briefly, in the Supreme Nudge trial, we implemented nudging and pricing strategies in supermarkets to promote healthy food purchases. In addition, a smartphone physical activity coaching app and a step counting app were randomised on the individual level across all supermarket clusters with the aim to increase daily step counts by providing this dynamically tailored coaching content. Participants in the physical activity intervention group received the step counter app plus a mobile coaching app sending messages, and participants in the control group received only the step counter app. Our target sample size was to include at least 360 participants to secure sufficient power for evaluation of the primary trial endpoint [26].

Twelve supermarkets of a Dutch supermarket chain within socially disadvantaged neighbourhoods participated in the trial. Supermarkets were located in relatively rural areas across different towns in the Netherlands. A socially disadvantaged neighbourhood was defined based on a below national average socioeconomic status score as calculated by The Netherlands Institute for Social Research [28]. The status scores were based on average levels of education, income and employment per postal code. The intervention implementation duration was 6 months to 1 year, depending on the supermarket enrolment date (further explained below). Participants were recruited from socially disadvantaged areas surrounding the participating supermarkets, and further eligibility criteria included: being between 30 and 80 years of age, being a regular shopper at a participating supermarket (> 50% of weekly household groceries purchased at participating supermarket), having the intention to continue visiting the supermarket for the next (half) year and being able to communicate in the Dutch language adequately. Additional individual-level randomisation in the smartphone physical activity intervention required participants to use a smartphone with a mobile data plan and Android 8, iOS 13 or more recent software versions installed, to have experience with mobile text messaging and to have no contra-indication for light physical activity. Follow-up measurements took place after 3 months, 6 months, and, depending on supermarket enrolment date, 12 months.

Participant recruitment and data collection for the trial were initially planned to take place in face-to-face appointments with participants. However, the COVID-19 pandemic forced us to adapt our protocols to a largely remote recruitment and data collection approach [26].

### Recruitment strategies

A stepwise recruitment strategy was applied, starting with a set of passive strategies followed by active strategies. The recruitment period ran from mid-January until November 2021 (Additional file 1: Supplementary Table 1). Passive strategies used first were news articles in local (online) media and flyers distributed in the supermarket and via mail. Next, Facebook posts were placed on the participating supermarket pages, posters were displayed in-store and at some other relevant locations in the neighbourhood (e.g. physiotherapy practices) and postal invitation letters were sent to every household of the municipality around the included supermarkets. Thereafter, the supermarket’s customer panels were sent an email invitation, a municipality-targeted social media campaign was launched (Facebook and Instagram) and an advertisement was placed on the website of the Dutch Heart Foundation (study funder). Moreover, two active recruitment approaches were used in addition to the passive strategies. The first was to encourage included participants via phone to ask their partner or neighbours to register for screening (promoting word-of-mouth), and the second was recruitment in the supermarket by researchers. As recruitment phases took place during 10 weeks in the spring and autumn of 2021, COVID-19-related restrictions were in place with varying intensity; a national lockdown was in effect at the start of the recruitment period, which was loosened by late spring. Therefore, the active in-store recruitment by the research team was conducted only when the restrictions allowed this—approximately halfway through the recruitment period. The recruitment materials mentioned that participants would receive a free grocery box after successful study completion.

### Data collection

The incurred costs and total staff hours needed for each recruitment strategy were logged by the research team in spread sheets. Total material cost (Euros) consisted of the sum of material costs spent on paper, envelopes and mailing costs of the recruitment letters or for example travel costs for in-store recruitment. Our supermarket partner helped with the design of the flyers, which was free of cost for the research team. Total time spent (hours) by research staff was calculated as the sum of all staff hours, which consisted of a combination of (unpaid) research interns and (paid) research assistants and pre-doctoral research fellows. All costs were made during the recruitment period. Information on participant registrations, eligibility, inclusion rates and measurement completion were centralised via a cloud-based clinical data management platform (Castor Electronic Data Capture). The screening questionnaire included an item asking participants how they had heard about the trial. Participants could provide multiple answers based on a pre-defined list describing all applied recruitment strategies, plus an open-ended answering option.

Data were self-reported and self-assessed by participants at home, considering the COVID-19-related restrictions. Data collection and study procedures are reported in a protocol paper [26]. This paper also reports in detail the exact measurements at the different time points during follow-up of the trial. Briefly, via the screening and baseline questionnaires, data were collected on *participant characteristics* including sex (male, female), age, highest completed educational attainment (here categorised as follows: low educational attainment: no education and primary education, medium educational attainment: secondary educational attainments, high educational attainment: tertiary educational attainments), household size (number of adults, number of children) and smoking status (current, irregular, former, never). In addition, questionnaire items on *medical history* asked participants about prevalent type 2 diabetes, hypertension, hyperlipidaemia and cardiovascular diseases and about medication use for these health conditions.

The primary trial outcome was *adherence to the Dutch dietary guidelines*, measured via the Dutch Healthy Diet 2015 food frequency questionnaire. Adherence to the Dutch dietary guidelines could range from 0, reflecting no adherence, to 150, reflecting full adherence (DHD15-index scores) [29]. Secondary outcomes were cardiometabolic risk markers, daily step counts, questionnaire items related to lifestyle behaviours and healthy food purchases, which are all further detailed below.

*Cardiometabolic risk markers* comprised of waist circumference (cm), HbA1c (mmol/mol), LDL-cholesterol (mmol/L), HDL-cholesterol (mmol/L), total cholesterol (mmol/L), total cholesterol/HDL-ratio and triglycerides (mmol/L). At-home measurement of cardiometabolic markers required participants to self-assess their waist circumference and perform a finger prick to collect capillary blood into two small capillary tubes. Instructional materials for the at-home measurements included a step-by-step instruction letter and a web-based video. The blood samples were sent back to the hospital lab via a medical return envelope in the mail, which took on average one day before the sample arrived in the lab. Results were analysed by enzymatic colorimetric test via the Roche/Hitachi Cobas C systems. In contrast to HbA1c, blood lipids remain less stable at room temperature (i.e. during mailing of the sample). As such, more invalid measurements of lipid profiles were expected. When the lab indicated the blood sample was invalid, participants were invited to collect another blood sample up to a maximum of three attempts. Research staff conducted home visits for participants requesting assistance with the cardiometabolic measurements as soon as COVID-19 restrictions allowed.

Data collection regarding the *healthy food purchases* were based on a supermarket loyalty card which participants were instructed to use during each supermarket visit for the complete study period. A baseline measurement for this outcome was defined as an average total percentage of healthy food and beverage purchases during a 4-week pre-intervention period, for those participants who started using a loyalty card during this period.

*Daily step counts* were measured via a step counter app that was developed as part of the Supreme Nudge app-based physical activity coaching system (SNapp). The development process of SNapp is described in greater detail elsewhere [30]. In brief, the app continuously quantifies step count using the smartphone’s built-in pedometer or accelerometer. Every hour, the app synchronises with a database server to store the user’s current step count level, date, time and type of sensor used for step count tracking (i.e. either a pedometer or accelerometer). The app is compatible with Android and iOS devices. Participants were asked to install the app based on textual and video instructions and to run the app on their personal smartphones during the Supreme Nudge trial. Participants received written instructions on how to use the app and were encouraged to carry their phones on their bodies as much as possible throughout the day. For the purpose of this study, the number of steps taken per day was averaged over a 7-day baseline period. We conducted a small validation study to explore whether the step counts derived from our app corresponded to step counts derived from default smartphone step counters (e.g. Samsung Health or the iOS Health app) and to step counts derived from accelerometers. We asked a convenience sample of 20 participants to wear GT3X ActiGraphs for at least 7 days and to report their wear time (start- and end time) in a diary. We also asked them to install our step counter app and, if available, report daily step count derived from their smartphones’ default step counters. Eleven participants had sufficient data available (≥ 4 days of GT3X ActiGraph accelerometer wear time and ≥ 4 days of reported step counts based on their default smartphone step counter). The Spearman’s correlation coefficient comparing the step counts derived from our app with those derived from default smartphone step counters was 0.62. The Spearman’s correlation coefficient comparing the step counts derived from our app with those derived from the accelerometers was 0.66. Based on these results, we considered our app to perform acceptable in counting steps in order to serve our research purposes.

Questionnaire data related to lifestyle behaviours were *food decision styles*, including reflective [31], habitual [32, 33] and impulsive [31, 34, 35] decision styles, *nudges and social cognitive factors* which measured health goals [36, 37], healthy shopping [38, 39], perceived social norm [40] and attractiveness of healthy foods [41], *customer satisfaction* measured via one general satisfaction score and eight sub-components and walking behaviours and social cognitive factors [42, 43]. All these questionnaire items were measured on 7-point Likert scales, ranging from fully disagree (1) to fully agree (7).

### Study outcomes and statistical analyses

The material costs, feasibility and yield of used recruitment strategies were assessed via descriptive statistics. Descriptive statistics report the total material cost (Euros) and total time spent (hours) by research staff for each applied recruitment strategy. In addition, the number and percentage (*n*(%)) of participants, the material costs, and hours spent per recruitment strategy are presented for (a) registered participants who completed eligibility assessment, (b) eligible participants, (c) included participants in the cluster-randomised trial, and (d) participants who completed the baseline measurement for the primary study outcome. Calculations of costs and hours spent per strategy are based on the total costs/hours per strategy divided by the number of participants (a) registered, (b) eligible, (c) included and (d) completed the baseline measurement for the primary trial outcome per recruitment strategy. All hours are calculated including travel time starting from the research office. Moreover, percentages are presented for the proportion of participants who indicated to have been recruited via one or two or more strategies.

Participant baseline characteristics and baseline measurements on trial outcomes are presented for the total sample. Continuous variables are described by the mean and standard deviation (SD) for normally distributed variables or by the median and interquartile range (IQR) for non-normally distributed variables. Categorical variables are presented by their number and percentage. Participant characteristics were explored stratified by supermarket location for visual inspection of potential differences in participant characteristics. Considering the physical activity intervention was randomised and implemented at the individual level, baseline characteristics and step count data for the control versus intervention participants of the physical activity coaching app intervention are presented in a separate baseline table.

To explore potential sociodemographic differences (age, sex, and educational attainment) within the top five most effective recruitment strategies, we assessed associations between the recruitment strategies (independent variables) and the sociodemographic variables (dependent variables) using linear multilevel models for age and logistic multilevel models for sex and educational attainment. All models included a random intercept on the supermarket level. Males were used as reference category in the analyses of sex, and the combination of low and medium educational attainment was used as reference category for the analyses of educational attainment. Participants with low and medium education were combined in one reference category due to very low participant numbers in the low educated group (< 10 participants for five out of the ten investigated outcomes). Moreover, we provided insight into the feasibility and costs of at-home measurement of cardiometabolic risk by reporting the related costs in terms of travel costs and staff hours for home visiting. Travel costs (Euros) and staff hours were calculated as the sum of all costs and staff hours related to the home visits. Furthermore, descriptive statistics are presented for the number and percentage (*n*(%)) of participants who (a) had a failed first attempt at the blood measurement and were invited for a second attempt, (b) ultimately did not complete the HbA1 and LDL measurement after a maximum of two attempts and (c) indicated to drop-out of the study due to experienced difficulties with the at-home measurements. Last, using the same analytical approach as for exploring sociodemographic differences in recruitment strategies, we explored potential sociodemographic differences in participants who required assistance via a home visit to complete the at-home measurement of cardiometabolic risk; who had a failed first attempt at the blood measurements or who were ultimately non-completers of the at-home measurement of HbA1c, LDL-cholesterol and/or waist circumference. Successful completion of cardiometabolic measurements was defined as completing a valid measurement result, after one or multiple attempts.

We presented regression coefficients and 95% confidence intervals to demonstrate the variation in recruitment effectiveness and at-home measurements across sociodemographic subgroups. We do not describe whether associations were statistically significant, because of the low numbers in some of the analysed subgroups. All analyses were conducted in R (version 4.0.3) using the packages *table1* [44] and *lme4* [45].

## Results

### Recruitment costs, yield and differential effectiveness per strategy

**Fig. 1:**
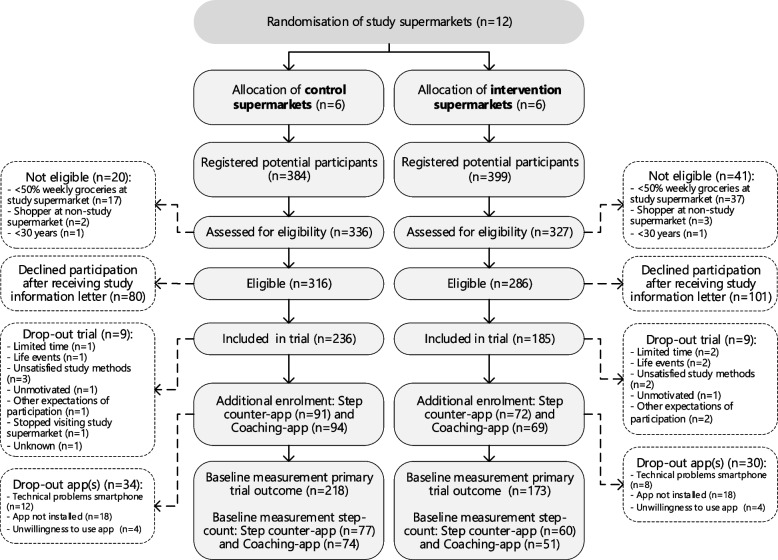
Participant flowchart of the Supreme Nudge trial

Figure 1 presents the participant flowchart up to baseline measurement completion of the primary trial outcome. In total, 783 individuals registered for participation, 602 (77%) were eligible and 421 (54%) were included as study participants after completing informed consent. The majority of participants who completed the eligibility assessment indicated to have been recruited via a single strategy (83%). Additionally, 16% indicated to have been recruited via two methods and 1% reported to have been exposed to three or more recruitment strategies. Most of the included participants (*n* = 421) were recruited via letters/flyers received at home (*n* = 314; 75%) or flyers received at the supermarket (*n* = 52; 12%) (Table 1). Less frequent reported strategies were in-store recruitment by a researcher (*n* = 32; 8%), news items (*n* = 31; 7%), word-of-mouth (*n* = 24; 6%) and social media (*n* = 16; 4%). Of paid strategies, flyers in the supermarket were the cheapest per participant included through this method (12 Euros) and the least time-invasive for research staff (< 1 h), whilst the most expensive strategies were the social media campaigns (194 Euros) and mailing of letters/flyers (89 Euros). In-store recruitment was high in terms of staff hours per included participant (9 h) as well as costs (50 Euros) (Table 1). A detailed breakdown of the costs per recruitment strategy is provided in Supplementary Table 2 (Additional file 1).

**Table 1:** Overview of recruitment investments and yields in the Supreme Nudge trial

			Completed eligibly assessment (*n* = 664)				Eligible (*n* = 602)				Included (*n* = 421)				Baseline measurement completion of primary trial outcome (*n* = 391)			
	Total material cost (€)	Total time (hours)	*n*	(%)	Material cost per participant (€)	Time per participant (hours)	*n*	(%)	Material cost per participant (€)	Time per participant (hours)	*n*	(%)	Material cost per participant (€)	Time per participant (hours)	*n*	(%)	Material cost per participant (€)	Time per participant (hours)
Media news article	0	10	49	(7.4)	0.0	0.2	44	(7.3)	0.0	0.2	31	(7.4)	0.0	0.3	31	(7.9)	0.0	0.3
Flyers in the supermarket	640	22	79	(11.9)	8.1	0.3	71	(11.8)	9.0	0.3	52	(12.4)	12.3	0.4	52	(13.3)	12.3	0.4
Posters	290	22	18	(2.7)	16.1	1.2	15	(2.5)	19.3	1.5	11	(2.6)	26.4	2.0	10	(2.6)	29.0	2.2
Mailing of flyers and recruitment letters	27,981	1120	477	(71.8)	58.7	2.4	433	(71.9)	64.6	2.6	314	(74.6)	89.1	3.6	288	(73.7)	96.5	3.9
Social media advertisements	3100	10	27	(4.1)	114.8	0.4	24	(4.0)	129.2	0.4	16	(3.8)	193.8	0.6	15	(3.8)	206.7	0.7
Email to supermarket customer panel	0	2	16	(2.4)	0.0	0.1	14	(2.3)	0.0	0.1	11	(2.6)	0.0	0.2	11	(2.8)	0.0	0.2
Advertisement on website of the Dutch Heart Foundation	0	2	17	(2.6)	0.0	0.1	16	(2.7)	0.0	0.1	12	(2.9)	0.0	0.2	11	(2.8)	0.0	0.2
Word of mouth	0	8	34	(5.1)	0.0	0.2	32	(5.3)	0.0	0.3	24	(5.7)	0.0	0.3	24	(6.1)	0.0	0.3
In-store recruitment	1583	276	68	(10.2)	23.3	4.1	67	(11.1)	23.6	4.1	32	(7.6)	49.5	8.6	30	(7.7)	52.8	9.2
Total	33,594	1472																

### Baseline characteristics of the included study sample

We enrolled 421 participants out of which 18 dropped out of the study before completing any baseline measurements and two never performed baseline measurements (Fig. 1). As such, baseline data were collected from 401 study participants, of which ten did not provide data for the primary study outcome and were therefore excluded, leaving 391 participants in the baseline sample. A total of 326 participants were randomised in the physical activity app intervention. Of those participants, 64 were excluded before the baseline period because they (1) never installed the step counter app, despite having received three reminders (*n* = 36; 56%), (2) used a smartphone that was incompatible with the step counter app (*n* = 20; 31%) or (3) were unwilling to install the step counter app (*n* = 8; 13%).

Trial participants who completed the measurement of the primary trial outcome (*n* = 391) had a mean age of 57.6 (SD 11.0) years, 71% (*n* = 282) were female and 41% (*n* = 162) were highly educated (Table 2). The population characteristics were approximately equally distributed across supermarket clusters, although some clusters included relatively more participants with high educational attainment than others (Additional file 1: Supplementary Table 3). On average, participants scored 104.7 (SD18.6) points on the DHD15-index (Table 2).

**Table 2:** Participant characteristics of the Supreme Nudge trial and baseline measures of outcomes, for the total sample (*n* = 391)

	Total sample (*n* = 391)	
**Population characteristics**		
Age, years, mean (SD)	57.7	(11.0)
Female^a^, *n* (%)	282	(72.1)
*Educational attainment, n (%)*		
Low	96	(24.6)
Medium	133	(34.0)
High	162	(41.4)
Household size, *n* adults^a^ (median [IQR])	2	[1]
Household size, *n* children^a^ (median [IQR])	0	[1]
*Smoking status*^*a*^*, n (%)*		
Current smoker	22	(5.6)
Irregular smoker	10	(2.5)
Former smoker	184	(46.8)
Never smoked	173	(44.0)
Prevalent type 2 diabetes^a^, *n* (%)	24	(6.1)
Medication for type 2 diabetes^a^, *n* (%)	23	(5.9)
Prevalent hypertension^a^, *n* (%)	60	(15.3)
Medication for hypertension^a^, *n* (%)	77	(19.6)
Prevalent hyperlipidaemia^a^, *n* (%)	55	(14.0)
Medication for hyperlipidaemia^a^, *n* (%)	55	(14.0)
Prevalent cardiovascular disease^a^, *n* (%)	46	(11.7)
**Primary outcome**		
Total dietary guideline adherence, scored 0 (low adherence) to 150 (high adherence), mean (SD)	104.7	(18.6)
**Secondary outcomes**		
*Dietary guideline adherence on sub-components, scored 0 (low adherence) to 10 (high adherence), mean (SD) or median [IQR]:*		
Vegetables	6.1	(3.2)
Fruits	6.7	(3.4)
Whole grains	7.3	(2.8)
Legumes	8.7	[10.0]
Nuts	5.8	[8.1]
Dairy	5.8	[6.5]
Fish	6.2	(3.5)
Cooking fats and butter	10.0	[9.7]
Tea	5.0	[9.8]
Coffee	7.8	(2.7)
Red meat	8.9	(2.6)
Processed meat	5.5	[6.3]
Sugar sweetened beverages	8.0	(3.2)
Alcoholic beverages	8.1	(3.3)
Salt	8.3	(2.1)
*Cardiometabolic measures, mean (SD):*		
HbA1c^b^, mmol/mol	37.4	(7.3)
LDL-cholesterol^c^, mmol/L	3.0	(1.0)
HDL-cholesterol ^d^, mmol/L	1.6	(0.5)
Total cholesterol^e^, mmol/L	5.4	(1.1)
Total cholesterol/HDL-ratio^e^	3.7	(1.2)
Triglycerides^f^, mmol/L	1.8	(1.0)
*Waist circumference*^*g*^*, cm, mean (SD):*		
Females	94.0	(13.7)
Males	103.0	(10.9)
*Healthy food purchases*^*h*^		
Total percentage healthy purchases	46.8	(22.3)
*Customer satisfaction*^*i*^*, scored 1 (low) to 7 (high), mean (SD):*		
Total customer satisfaction	5.6	(1.1)
Environment and atmosphere	5.6	(1.2)
Layout and routing	5.5	(1.1)
Supermarket tidiness	5.8	(1.1)
Assortment of food products	5.2	(1.2)
General product prices	5.0	(1.2)
Discount prices	5.6	(1.0)
Fruit and vegetable prices	5.0	(1.3)
Bread prices	5.2	(1.2)
*Food decision style for vegetables*^*j*^*, scored 1 (low) to 7 (high), mean (SD):*		
Reflective	5.2	(1.1)
Habitual	5.0	(0.9)
Impulsive	3.5	(1.2)
*Food decision style for snacks*^*k*^*, scored 1 (low) to 7 (high), mean (SD):*		
Reflective	4.0	(1.4)
Habitual	3.1	(1.4)
Impulsive	3.6	(1.6)
*Nudges and social cognitive factors*^*i*^*, scored 1 (low) to 7 (high), mean (SD):*		
Health goals	6.3	(0.9)
Healthy shopping	6.0	(1.0)
Perceived social norm	4.7	(1.0)
Attractiveness healthy foods	5.9	(1.1)

Low educational attainment: no education and primary education; medium educational attainment: secondary educational attainments; high educational attainment: tertiary educational attainments.^a^*n* = 2 missing values; ^b^*n* = 22 missing values; ^c^*n* = 49 missing values; ^d^*n* = 36 missing values; ^e^*n* = 48 missing values; ^f^*n* = 43 missing values; ^g^*n* = 6 missing values; ^h^*n* = 161 missing values; ^i^*n* = 3 missing values; ^j^*n* = 30 missing values; ^k^*n* = 139 missing values

Table 3 presents the baseline and demographic characteristics of the participants in the physical activity app intervention. A total of 137 participants were randomly assigned to the control group and 125 participants to the intervention group. All these participants completed the baseline measurement of step counts. Participants (mean age 56.6 years (SD 11.0); *n* = 195 (74%) females; *n* = 113 (43%) high educated) mostly used Android smartphones (*n* = 184/262, 70%) and walked a median of 2710 (IQR 3696) steps per day during the 7-day baseline period. Daily step counts at baseline were equally balanced between the control and intervention groups.

**Table 3:** Participant characteristics and baseline measurement of step counts for Supreme Nudge trial participants additionally included in the physical activity coaching app intervention, for the total sample (*n* = 262)

	Total sample physical activity intervention (*n* = 262)	
Age, years (mean (SD))	56.5	(11.0)
Female (*n* (%))	195	(74.4)
*Educational attainment (n (%))*		
Low	50	(19.1)
Medium	99	(37.8)
High	113	(43.1)
*Smartphone type:*		
Android, *n* (%)	184	(70.2)
iOS, *n* (%)	78	(29.8)
*Daily step count, median [IQR]:*		
Averaged over 7-day baseline period	2710	[3696]
*Walking behaviour and social cognitive factors*^a^*, scored 1–7, mean (SD)*		
Consequences of behaviour	3.1	(2.0)
Social comparison	2.6	(1.7)
Action planning	4.0	(1.9)
Self-monitoring	3.3	(1.9)
Social support	2.5	(1.7)
Goal setting	3.4	(1.9)
Barrier identification	2.2	(1.6)
Self-evaluation	3.7	(1.9)
Encouragement	2.5	(1.8)
Others’ approval	1.7	(1.3)
eHealth literacy^a^, scored 1–7, mean (SD)	4.9	(1.5)

Low educational attainment: no education and primary education; medium educational attainment: secondary educational attainments; high educational attainment: tertiary educational attainments.^a^*n* = 19 missing values

### Sociodemographic differences in effectiveness of recruitment strategies

Flyers in the supermarket (*β* 2.56 years (95% CI − 0.63; 5.85)) and media news articles (*β* 2.33 years (95% CI − 1.69; 6.44)) recruited somewhat older participants and relatively more females (flyers: OR_females_ 1.91 (95% CI 0.93; 4.31) and news articles: OR_females_ 1.64 (95% CI: 0.69; 4.51)). Word-of-mouth tended to recruit relatively more males (OR_females_ 0.51 (95% CI 0.22; 1.21)). Those with low and medium educational attainment tended to be more often recruited via flyers in the supermarket (OR_high education_ 0.72 (95% CI 0.38; 1.33)) and news articles (OR_high education_ 0.67 (95% CI 0.29; 1.45)), whilst those with high educational attainment via in-store recruitment (OR_high education_ 1.75 (95% CI 0.81; 3.81)) (Table 4). Descriptive statistics and the absolute numbers analysed per group are presented in Supplementary Table 4 (Additional file 1).

**Table 4:** Associations between the top five most successful recruitment strategies and the sociodemographic variables age, sex (female) and educational attainment (high education) (*n* = 391)

	Age^a^, years		Sex		Educational attainment	
	*β*	(95% CI)	OR_females_	(95% CI)	OR_high education_	(95% CI)
Recruited via mailing of recruitment flyers and letters (*n* = 288)	− 1.76	(− 4.29; 0.70)	1.00	(0.60; 1.64)	1.22	(0.76; 1.98)
Recruited via flyers in the supermarket (*n* = 52)	2.56	(− 0.63; 5.85)	1.91	(0.93; 4.31)	0.72	(0.38; 1.33)
Recruited via media news article (*n* = 31)	2.33	(− 1.69; 6.44)	1.64	(0.69; 4.51)	0.67	(0.29; 1.45)
Recruited via in-store recruitment (*n* = 30)	− 0.11	(− 4.19; 4.06)	1.21	(0.52; 3.14)	1.75	(0.81; 3.81)
Recruited via word-of-mouth (*n* = 24)	− 0.40	(− 4.94; 4.14)	0.51	(0.22; 1.21)	1.01	(0.42; 2.35)

Statistical significant outcomes (*p* < 0.05) are displayed in bold text. Associations are based on linear multilevel models (age) and logistic multilevel models (sex and education), including a random intercept on the supermarket level. Males are used as reference category in the analyses of sex, and the combination of low and medium educational attainment was used as reference category for the analyses of educational attainment. *OR* odds ratio, *CI* confidence interval; ^a^*n* = 2 missing values

### Feasibility and sociodemographic differences in at-home measurement of cardiometabolic risk

At-home measurements were often completed successfully, as for lipid profile 88% (*n* = 342) of participants completed the measurement, 94% (*n* = 369) for HbA1c and 99% (*n* = 385) for waist circumference (Table 1). Researcher assistance with the at-home measurements was requested by 17% (*n* = 68) of the total sample. On average, the time spent per participant by research staff was almost 2 h (including preparation time and travel time), and the accompanying travel costs via a shared rental car were 24 Euros, resulting in 129 staff hours and 1672 Euros travel costs in total during the baseline measurements attributable to conducting home visits. A first attempt at the blood measurements failed for 86 participants (22%), who were invited for a second attempt. Ultimately, the HbA1c measurement was not completed by 20 participants (5%) and the LDL measurement by 47 participants (12%). Three participants (< 1%) dropped out of the study due to persisting difficulties with the at-home measurements.

Those with a failed first attempt at the blood measurement were relatively more often lower educated (OR_high education_ 0.64 (95% CI 0.38; 1.06)) and relatively older (*β* 3.89 years (95% CI 1.28; 6.49), whilst the non-completers of the HbA1c (*β* − 8.92 years (95% CI − 13.62; − 4.28)) and LDL (*β* − 3.19 years (95% CI − 6.53; 0.09)) were relatively younger. Among non-completers of the waist circumference measurement were relatively more males than females (OR_females_ 0.18 (95% CI 0.03; 0.95) (Table 5). Descriptive statistics and the absolute numbers analysed per group are presented in Supplementary Table 5 (Additional file 1).

**Table 5:** Associations between elements of at-home measurement of cardiometabolic risk markers and the sociodemographic variables age, sex (female) and educational attainment (high education) (*n* = 391)

	Age^a^, years		Sex		Educational attainment	
	*β*	(95% CI)	OR_females_	(95% CI)	OR_high education_	(95% CI)
Participants requesting a home-visit (*n* = 68)	0.96	(− 1.93; 3.84)	0.97	(0.56; 1.78)	1.11	(0.64; 1.92)
Failed first attempt of blood measurement (*n* = 86)	**3.89**	(**1.28**; **6.49**)	1.13	(0.67; 1.99)	0.64	(0.38; 1.06)
Non-completers HbA1c measurement (*n* = 22)	**− 8.92**	(− **13.62**; **− 4.28**)	1.15	(0.43; 3.60)	1.46	(0.59; 3.58)
Non-completers LDL-cholesterol measurement (*n* = 49)	− 3.19	(− 6.53; 0.09)	0.79	(0.41; 1.55)	0.91	(0.48; 1.71)
Non-completers waist circumference measurement (*n* = 8)	− 0.61	(− 8.38; 7.04)	**0.18**	(**0.03**; **0.95**)	1.45	(0.33; 6.37)

Statistical significant outcomes (*p* < 0.05) are displayed in bold text. Associations are based on linear multilevel models (age) and logistic multilevel models (sex and education), including a random intercept on the supermarket level. Males are used as reference category in the analyses of sex, and the combination of low and medium educational attainment was used as reference category for the analyses of educational attainment. *OR* odds ratio, *CI* confidence interval; ^a^*n* = 2 missing values

## Discussion

This paper reports on costs, feasibility and results of used recruitment strategies, baseline characteristics of the study sample, potential sociodemographic differences in successful recruitment strategies and feasibility of cardiometabolic measurements at home. The results indicate that letters/flyers sent to home addresses and distributed in the supermarkets recruited most participants. Supermarket flyers were the cheapest and least time-invasive of all paid recruitment strategies. The social media campaign was the most expensive, and mailings of recruitment letters and in-store-recruitment were the most time-invasive and therefore costly in terms of recruited staff hours. Flyers in the supermarket and media news articles seemed to recruit relatively more females and word-of-mouth seemed to recruit relatively more males. At-home measurement of cardiometabolic markers was feasible, as they were successfully completed by most participants. Older participants had more often a failed first attempt of blood measurement, whilst younger participants were more often non-completers of the blood measurements.

The success of mailing letters and flyers to home addresses and the distribution of flyers in the supermarket is likely due to their wide reach and the fact that these strategies were used early in the recruitment process. The fact that our social media campaign, which was used later, appeared very costly in terms of recruitment yield is in contrast to what other studies showed. In studies where social media were used as the primary recruitment method, it appeared (cost)-effective (e.g. ~6–14 Euros per included participant [46, 47]). It is possible that many of our study participants had already registered early in the recruitment process before exposure to the social media campaign. The social media campaign may have persuaded those who were initially doubtful or forgot about the study after having first received a letter or flyer. The same could apply to in-store recruitment, as many shoppers had mentioned that they had seen a flyer but had not acted upon it. Although it is to be expected that participants registered after having been exposed to multiple recruitment strategies, only a few participants indicated to have been recruited via multiple strategies. This could be the result of underreporting due to recall bias or unconscious processing of exposure to recruitment strategies [48, 49].

The included study sample was comparable with the general Dutch population in terms of educational attainment, but females were overrepresented and participants scored relatively high on the DHD15-index. Overrepresentation of females in epidemiological studies is a well-known phenomenon [50]. This may be due to higher levels of health consciousness among females, a relatively higher interest in food-related topics, or having primary responsibility for grocery shopping within a household. Regarding the DHD15-index score, included participants scored relatively high with 105 points on average compared to, for example, a cross-sectional Dutch population sample in the *Eet & Leef* study (average of 96 points) [51]. This suggests that our study sample may be more health-conscious than the general Dutch population. Furthermore, our sample underrepresented smokers (~5% versus ~19% nationally) [52] but was representative in terms of the prevalence of hypertension [53].

Our results suggested that passive recruitment strategies yielded more females whilst active strategies more males, which is also seen in other studies [20, 46]. Specifically, our supermarket flyers and the media news articles seemed to have recruited relatively more females and also relatively older participants. This may partly be explained by a larger responsibility for grocery shopping or a higher frequency of supermarket visits, leading to larger exposure to the supermarket flyers. Also, that the active strategy word-of-mouth seemed to recruit more males is likely the result of encouraging included participants (mainly female) to ask their partner if they are interested to participate. Furthermore, our findings suggested that passive recruitment strategies recruited relatively more participants with low and medium educational attainment compared to active in-store recruitment, via which relatively more participants with high educational attainment were recruited. This is in contrast to another study which indicated that passive strategies often recruit relatively more individuals with high educational attainment compared to active strategies [20]. It could be that individuals with low and medium educational attainment were relatively more susceptible to the content of the flyers mentioning the free grocery box as an incentive.

The COVID-19 pandemic had a considerable impact on recruitment and data collection procedures. At-home measurement of cardiometabolic makers and self-installation of the coaching app may have been challenging for some participants, since participants were required to understand and be able to follow instructions from a written manual or video. Participants only received support from research staff when actively asking for it, which requires a certain level of self-efficacy. This may have led to some selection bias, resulting in a study sample consisting of participants with relatively high levels of self-efficacy. The at-home measurements may have introduced self-assessment bias and reduced measurement reliability for waist circumference measurement. Previous research has shown that especially those with lower educational attainment had relatively more often an overestimation of their waist circumference measurement, as well as younger versus older participants [54]. Another challenge was that the COVID-19 pandemic itself likely interfered with inclusion and the completion of measurements, as individuals could have been hesitant to register, unable to participate or complete measurements. By investing in sending out multiple at-home measurement packages and conducting home visits, we reached a relatively high completion rate of the blood measurements. Yet, blood measurements were not successfully completed for all participants, and reasons for non-completion of blood measurements were often practical (e.g. insufficient blood flow). Logistically, at-home cardiometabolic measurements were to some extent efficient, since this saved staff hours and costs due to limited commute and work at study locations. On the other hand, conducting home visits to aid with the cardiometabolic measurements was also costly and time-invasive.

We recruited participants in socially disadvantaged neighbourhoods via various passive and active recruitment approaches with the aim to recruit a relative high proportion of those with a lower and medium educational attainment [26]. Yet, despite these efforts, our sample consisted of 41% highly educated participants, and insights from this paper demonstrate that recruitment is challenging and costly. Another important insight was that the use of flyers/letters sent to home addresses can be an effective recruitment strategy, although it is very costly. Thus, the use of complementary recruitment strategies is recommended to boost inclusion rates, combined with strategies which could for example be promoting word-of-mouth to reach a specific group. Researchers may consider at-home cardiometabolic measurements to simplify logistics and reach potential participants from widespread geographical locations. Future studies with larger sample sizes could focus on further determining the most effective recruitment strategies to reach individuals with a low SEP and could investigate the importance of the order in which recruitment strategies are used on their (cost)-effectiveness. Since recruitment is time invasive and costly, it is recommended that future projects consider comprehensive recruitment plans and allocate sufficient time and financial resources for the recruitment process.

### Strengths and limitations

Strengths of this study are the utilisation of a wide range of recruitment strategies and the monitoring of used strategies and recruitment yields to explore their effects. Nevertheless, determination of the most decisive strategy for participants to register was not possible since participants were not asked for the most decisive method and had the option to register multiple strategies instead of just one. Also, the relatively small sample size when exploring sociodemographic differences in recruitment strategy effectiveness and cardiometabolic measurements likely led to wide confidence intervals. For the interpretation of the results, we therefore focused on interpretation of effect sizes rather than statistical significance. Moreover, this study did not investigate the influence of the order in which recruitment strategies were used and time intervals between recruitment strategies on their (cost)-effectiveness. Furthermore, since this study was performed during the COVID-19 pandemic, this may have affected recruitment yield, but we were unable to quantify this impact. Last, we lack sufficient insight into reasons why 181 eligible participants did not proceed with study enrolment, since most of them did not respond after having received the information letter. Some replied that participation appeared more comprehensive (e.g. with at-home blood measurements) than anticipated which may have been a barrier for others as well.

## Conclusion

In the Supreme Nudge trial, supermarket flyers were the most cost-effective in recruiting participants of all paid strategies. Mailings to home addresses recruited the most participants but were very costly. Conducting at-home cardiometabolic measurements was feasible with relatively high completion rates. These insights are valuable for future research projects that aim to recruit a diverse study population and to study cardiometabolic risk markers in, for example, geographically large and widespread groups or when face to face contact is not possible.

## Supplementary Information

**Additional file 1: Supplementary Table 1.** Recruitment strategy utilisation, per month in 2021. **Supplementary Table 2.** Detailed breakdown of material costs and of time per recruitment strategy. **Supplementary Table 3.** Population characteristics of the Supreme Nudge trial (n=391) presented by supermarket clusters (n=12). **Supplementary Table 4.** Absolute numbers analyse per group for the top five most successful recruitment strategies and the sociodemographic variables age, sex and educational attainment (n=391). **Supplementary Table 5.** Absolute numbers analysed for elements of at-home measurement of cardiometabolic risk markers and the sociodemographic variables age, sex and educational attainment (n=391).

## Data Availability

The data analysed during the current study are not publicly available as it will violate participant consent. The analysis plan and syntax are available from the corresponding author on reasonable request.
