# Supplementary material for: Participant recruitment, baseline characteristics and at-home-measurements of cardiometabolic risk markers: insights from the Supreme Nudge parallel cluster-randomised controlled supermarket trial
Source: Trials. 2023 Mar 2;24:159. doi: 10.1186/s13063-023-07157-8 (PMC9981252; doi:10.1186/s13063-023-07157-8)
Supplement: Supplementary file 1 — Additional file 1: Supplementary Table 1. Recruitment strategy utilisation, per month in 2021. Supplementary Table 2. Detailed breakdown of material costs and of time per recruitment strategy. Supplementary Table 3. Population characteristics of the Supreme Nudge trial (n=391) presented by supermarket clusters (n=12). Supplementary Table 4. Absolute numbers analyse per group for the top five most successful recruitment strategies and the sociodemographic variables age, sex and educational attainment (n=391). Supplementary Table 5. Absolute numbers analysed for elements of at-home measurement of cardiometabolic risk markers and the sociodemographic variables age, sex and educational attainment (n=391). [file 13063_2023_7157_MOESM1_ESM.docx]

**Participant recruitment, baseline** **characteristics** **and at-home-measurements of cardiometabolic risk markers: insights from the Supreme Nudge parallel cluster-randomised controlled supermarket trial**

*Josine M. Stuber & Beryl A.C.E. van Hoek, et al.*

Supplementary material

**Supplementary Table 1.** Recruitment strategy utilisation, per month in 2021.

|  |  |  | Spring 2021 |  |  |  |  | Autumn 2021 |  |
| --- | --- | --- | --- | --- | --- | --- | --- | --- | --- |
|  | January | February | Mach | April | May |  | September | October | November |
| Supermarkets #1 - #4 | - Local (online) media |  |  |  |  |  |  |  |  |
|  | - Flyers/letters to households |  |  |  |  |  |  |  |  |
|  | - Flyers and posters |  |  |  |  |  |  |  |  |
|  |  | - Post supermarket’s Facebook timeline |  |  |  |  |  |  |  |
|  |  | - Supermarket’s customer panel mailing |  |  |  |  |  |  |  |
|  |  | - Social media campaign |  |  |  |  |  |  |  |
|  |  |  | - Advertisement website study funder |  |  |  |  |  |  |
|  |  |  | - Promoting word-of-mouth |  |  |  |  |  |  |
|  |  |  | - In-store recruitment |  |  |  |  |  |  |
| Supermarkets #5 - #8 |  |  | - Local (online) media |  |  |  |  |  |  |
|  |  |  | - Flyers/letters to households |  |  |  |  |  |  |
|  |  |  | - Post supermarket’s Facebook timeline |  |  |  |  |  |  |
|  |  |  | - Flyers and posters |  |  |  |  |  |  |
|  |  |  |  | - Supermarket’s customer panel mailing |  |  |  |  |  |
|  |  |  |  | - Advertisement website study funder |  |  |  |  |  |
|  |  |  |  | - Social media campaign |  |  |  |  |  |
|  |  |  |  | - Promoting word-of-mouth | |  |  |  |  |
|  |  |  |  | - In-store recruitment |  |  |  |  |  |
| Supermarkets #9 - #12 |  |  |  |  |  |  | - Local (online) media |  |  |
|  |  |  |  |  |  |  | - Flyers/letters to households |  |  |
|  |  |  |  |  |  |  | - Post supermarket’s Facebook timeline |  |  |
|  |  |  |  |  |  |  | - Flyers and posters |  |  |
|  |  |  |  |  |  |  |  | - Supermarket’s customer panel mailing |  |
|  |  |  |  |  |  |  |  | - Advertisement website study funder |  |
|  |  |  |  |  |  |  |  | - Social media campaign |  |
|  |  |  |  |  |  |  |  | - Promoting word-of-mouth | |
|  |  |  |  |  |  |  |  | - In-store recruitment |  |

**Supplementary Table 2.** Detailed breakdown of material costs and of time per recruitment strategy

|  | Total material cost (€) | Cost (€) for paper/print materials | Cost (€) for mail | Cost (€) for straff travel | Cost (€) for use of service | Total time (hours) | Time (hours) for development of materials | Time (hours) for implementing/preparing materials |
| --- | --- | --- | --- | --- | --- | --- | --- | --- |
| Media news article | 0 | 0 | 0 | 0 | 0 | 10 | 6 | 4 |
| Flyers in the supermarket | 640 | 490 | 0 | 150 | 0 | 22 | 12 | 10 |
| Posters | 290 | 140 | 0 | 150 | 0 | 22 | 12 | 10 |
| Mailing of flyers and recruitment letters | 27,981 | 2535 | 25446 | 0 | 0 | 1120 | 5 | 1115 |
| Social media advertisements | 3100 | 0 | 0 | 0 | 3100 | 10 | 2 | 8 |
| Email to supermarket customer panel | 0 | 0 | 0 | 0 | 0 | 2 | 0 | 2 |
| Advertisement on website of the Dutch Heart Foundation | 0 | 0 | 0 | 0 | 0 | 2 | 0 | 2 |
| Word of mouth | 0 | 0 | 0 | 0 | 0 | 8 | 1 | 7 |
| In-store recruitment | 1,583 | 0 | 0 | 1583 | 0 | 276 | 1 | 275 |

**Supplementary Table 3.** Population characteristics of the Supreme Nudge trial (n=391) presented by supermarket clusters (n=12).

|  |  | Store #1 | Store #2 | Store #3 | Store #4 | Store #5 | Store #6 | Store #7 | Store #8 | Store #9 | Store #10 | Store #11 | Store #12 |
| --- | --- | --- | --- | --- | --- | --- | --- | --- | --- | --- | --- | --- | --- |
|  |  | (n=47) | (n =38) | (n=30) | (n=21) | (n=34) | (n=36) | (n=36) | (n=13) | (n=39) | (n=46) | (n=21) | (n=30) |
| Age, years (mean (SD)) | | 56.9 (8.6) | 61.1 (9.4) | 55.8 (10.0) | 56.5 (10.3) | 57.1 (14.0) | 60.1 (13.3) | 55.1 (9.4) | 56.9 (12.2) | 59.8 (9.9) | 58.4 (10.5) | 59.0 (11.9) | 53.9 (12.4) |
| *Educational attainment (n (%))* | |  |  |  |  |  |  |  |  |  |  |  |  |
| Low | | 6 (12.8) | 10 (26.3) | 7 (23.3) | 7 (33.3) | 7 (20.6) | 8 (22.2) | 6 (16.7) | 7 (53.8) | 10 (25.6) | 12 (26.1) | 8 (38.1) | 8 (26.7) |
| Medium | | 22 (46.8) | 16 (42.1) | 11 (36.7) | 9 (42.9) | 10 (29.4) | 7 (19.4) | 8 (22.2) | 3 (23.1) | 14 (35.9) | 14 (30.4) | 9 (42.9) | 10 (33.3) |
| High | | 19 (40.4) | 12 (31.6) | 12 (40.0) | 5 (23.8) | 17 (50.0) | 21 (58.3) | 22 (61.1) | 3 (23.1) | 15 (38.5) | 20 (43.5) | 4 (19.0) | 12 (40.0) |
| Female (n (%)) | | 36 (76.6) | 26 (68.4) | 22 (73.3) | 12 (57.1) | 27 (79.4) | 28 (77.8) | 27 (75.0) | 10 (76.9) | 28 (71.8) | 32 (69.6) | 13 (61.9) | 21 (70.0) |
| Household size, *n* adults (median [IQR]) | | 2.0 [0.0] | 2.0 [0.8] | 2.0 [1.0] | 2.0 [0.0] | 1.0 [1.0] | 2.0 [1.0] | 2.0 [1.0] | 2.0 [1.0] | 2.0 [1.0] | 2.0 [0] | 1.0 [1.0] | 1.0 [1.0] |
| Household size, *n* children (median [IQR]) | | 0.0 [1.0] | 0.0 [0] | 0.0 [1.0] | 0.0 [1.0] | 0.0 [1.0] | 0.0 [0.3] | 0.0 [1.0] | 0.0 [2.0] | 0.0 [0.0] | 0.0 [1.0] | 0.0 [0.0] | 0.0 [1.0] |
| *Smoking status (n(%))* | |  |  |  |  |  |  |  |  |  |  |  |  |
| Current smoker | | 3 (6.4) | 2 (5.3) | 2 (6.7) | 2 (9.5) | 1 (2.9) | 2 (5.6) | 1 (2.8) | 1 (7.7) | 2 (5.1) | 0 (0.0) | 4 (19.0) | 2 (6.7) |
| Irregular smoker | | 3 (6.4) | 0 (0.0) | 0 (0.0) | 0 (0.0) | 3 (8.8) | 1 (2.8) | 1 (2.8) | 0 (0.0) | 0 (0.0) | 1 (2.2) | 1 (4.8) | 0 (0.0) |
| Former smoker | | 23 (48.9) | 13 (34.2) | 14 (46.7) | 10 (47.6) | 11 (32.4) | 17 (47.2) | 18 (50.0) | 8 (61.5) | 17 (43.6) | 26 (56.5) | 10 (47.6) | 17 (56.7) |
| Never smoked | | 18 (38.3) | 23 (60.5) | 13 (43.3) | 9 (42.9) | 19 (55.9) | 16 (44.4) | 16 (44.4) | 4 (30.8) | 20 (51.3) | 19 (41.3) | 6 (28.6) | 10 (33.3) |
| Prevalent type 2 diabetes (n(%)) | | 3 (6.4) | 5 (13.2) | 3 (10.0) | 5 (23.8) | 0 (0.0) | 1 (2.8) | 2 (5.6) | 1 (7.7) | 2 (5.1) | 0 (0.0) | 1 (4.8) | 1 (3.3) |
| Medication for type 2 diabetes (n(%)) | | 3 (6.4) | 5 (13.2) | 3 (10.0) | 4 (19.0) | 0 (0.0) | 1 (2.8) | 2 (5.6) | 1 (7.7) | 2 (5.1) | 0 (0.0) | 1 (4.8) | 1 (3.3) |
| Prevalent hypertension (n(%)) | | 6 (12.8) | 12 (31.6) | 8 (26.7) | 3 (14.3) | 0 (0.0) | 6 (16.7) | 1 (2.8) | 2 (15.4) | 8 (20.5) | 7 (15.2) | 2 (9.5) | 5 (16.7) |
| Medication for hypertension (n(%)) | | 7 (14.9) | 13 (34.2) | 7 (23.3) | 3 (14.3) | 6 (17.6) | 8 (22.2) | 2 (5.6) | 2 (15.4) | 9 (23.1) | 10 (21.7) | 2 (9.5) | 8 (26.7) |
| Prevalent hyperlipidaemia (n(%)) | | 6 (12.8) | 3 (7.9) | 8 (26.7) | 4 (19.0) | 2 (5.9) | 7 (19.4) | 5 (13.9) | 1 (7.7) | 8 (20.5) | 7 (15.2) | 3 (14.3) | 1 (3.3) |
| Medication for hyperlipidaemia (n(%)) | | 7 (14.9) | 5 (13.2) | 3 (10.0) | 3 (14.3) | 4 (11.8) | 7 (19.4) | 3 (8.3) | 2 (15.4) | 5 (12.8) | 7 (15.2) | 3 (14.3) | 6 (20.0) |
| Prevalent cardiovascular disease (n(%)) | | 5 (10.6) | 5 (13.2) | 1 (3.3) | 4 (19.0) | 4 (11.8) | 6 (16.7) | 1 (2.8) | 0 (0.0) | 5 (12.8) | 5 (10.9) | 5 (23.8) | 5 (16.7) |

Low educational attainment: no education and primary education; Medium educational attainment: secondary educational attainments; High educational attainment: tertiary educational attainments.

**Supplementary Table 4.** Absolute numbers analyse per group for the top five most successful recruitment strategies and the sociodemographic variables age, sex and educational attainment (n=391).

|  | Age^a^, years | Sex | | Educational attainment | | |
| --- | --- | --- | --- | --- | --- | --- |
|  | Mean (SD) | Females, n (%) | Males, n (%) | Low, n (%) | Medium, n (%) | High, n (%) |
| Recruited via mailing of recruitment flyers and letters (n= 288) | 57.2 (11.2) | 208 (72.5) | 79 (27.5) | 64 (22.2) | 100 (34.7) | 124 (43.1) |
| Recruited via flyers in the supermarket (n=52) | 60.0 (10.6) | 42 (82.4) | 9 (17.7) | 18 (34.6) | 16 (30.8) | 18 (34.6) |
| Recruited via media news article (n=31) | 60.0 (10.2) | 25 (80.7) | 6 (19.4) | 9 (29.0) | 12 (38.7) | 10 (32.3) |
| Recruited via in-store recruitment (n=30) | 57.8 (12.8) | 22 (75.9) | 7 (24.1) | 4 (13.3) | 10 (33.3) | 16 (53.3) |
| Recruited via word-of-mouth (n=24) | 57.3 (10.5) | 14 (58.3) | 10 (41.7) | 7 (29.2) | 7 (29.2) | 10 (41.7) |

Low educational attainment: no education and primary education; Medium educational attainment: secondary educational attainments; High educational attainment: tertiary educational attainments. ^a^n=2 missing values.

**Supplementary Table 5.** Absolute numbers analysed for elements of at-home measurement of cardiometabolic risk markers and the sociodemographic variables age, sex and educational attainment (n=391).

|  | Age^a^, years | Sex | | Educational attainment | | |
| --- | --- | --- | --- | --- | --- | --- |
|  | Mean (SD) | Females, n (%) | Males, n (%) | Low, n (%) | Medium, n (%) | High, n (%) |
| Participants requesting a home-visit (n=68) | 58.5 (11.8) | 49 (72.1) | 19 (27.9) | 21 (30.9) | 18 (26.5) | 29 (42.6) |
| Failed first attempt of blood measurement (n=86) | 60.7 (10.0) | 64 (74.4) | 22 (25.6) | 29 (33.7) | 28 (32.6) | 29 (33.7) |
| Non-completers HbA1c measurement, (n=22) | 49.2 (12.1) | 15 (75.0) | 5 (25.0) | 6 (27.3) | 5 (22.7) | 11 (50.0) |
| Non-completers LDL-cholesterol measurement (n=49) | 54.8 (12.7) | 32 (68.1) | 15 (31.9) | 16 (32.7) | 13 (26.5) | 20 (40.8) |
| Non-completers waist circumference measurement (n=8) | 56.9 (9.0) | 2 (33.3) | 4 (66.7) | 3 (37.5) | 1 (12.5) | 4 (50.0) |

Low educational attainment: no education and primary education; Medium educational attainment: secondary educational attainments; High educational attainment: tertiary educational attainments. ^a^n=2 missing values.
